# Postinfluenza Vaccination Idiopathic Thrombocytopenic Purpura in Three Elderly Patients

**DOI:** 10.1155/2016/7913092

**Published:** 2016-02-21

**Authors:** Joji Nagasaki, Masahiro Manabe, Kentaro Ido, Hiroyoshi Ichihara, Yasutaka Aoyama, Tadanobu Ohta, Yoshio Furukawa, Atsuko Mugitani

**Affiliations:** Department of Hematology, Seichokai Fuchu Hospital, 1-10-17 Hikocho, Izumi-shi, Osaka 594-0076, Japan

## Abstract

The etiologies of secondary idiopathic thrombocytopenic purpura (ITP) include infection, autoimmune disease, and immunodeficiency. We report the cases of three elderly patients who developed ITP after receiving influenza vaccinations. The platelet count of an 81-year-old woman fell to 27,000/*μ*L after she received an influenza vaccination. A 75-year-old woman developed thrombocytopenia (5,000 platelets/*μ*L) after receiving an influenza vaccination. An 87-year-old woman whose laboratory test values included a platelet count of 2,000/*μ*L experienced genital bleeding after receiving an influenza vaccination. After* Helicobacter pylori* (HP) eradication or corticosteroid treatment, all of the patients' platelet counts increased. Influenza vaccination is an underlying etiology of ITP in elderly patients. HP eradication or corticosteroid treatment is effective for these patients. Clinicians should be aware of the association between ITP and influenza vaccinations.

## 1. Introduction

Idiopathic thrombocytopenic purpura (ITP) is an acquired form of thrombocytopenia caused by autoantibody-mediated platelet destruction. The exact cause of ITP is unknown. It is initially treated with corticosteroids, intravenous immunoglobulin (IVIG), or intravenous anti-D immunoglobulin (IV anti-D). Corticosteroids are commonly recognized as a first-line therapy for acute ITP in adult patients. IVIG and IV anti-D are used for patients who need a rapid platelet increase or have a contraindication for corticosteroids. Although these treatments are effective and indispensable, the existence of treatment-related adverse events should be noted, especially in elderly patients. Here we report three elderly patients with ITP who had recently received influenza vaccinations. Their platelet counts increased after eradication of* Helicobacter pylori* (HP) or treatment with a corticosteroid.

## 2. Case Reports

### 2.1. Case 1

An 81-year-old woman whose platelet count was 184,000/*μ*L 3 months before admission was referred to our hospital because of thrombocytopenia. Her platelet count reduction was found unexpectedly during a medical examination; she had no symptoms caused by thrombocytopenia. She had received an influenza vaccination 4 weeks before developing thrombocytopenia. Her laboratory data at the time of thrombocytopenia diagnosis were as follows: platelet count, 39,000/*μ*L; white blood cell count, 4,900/*μ*L without atypical cells; hemoglobin concentration, 14.1 g/dL; and platelet-associated IgG (PAIgG), 97 ng/10^7^ cells. Bone marrow was normocellular; the megakaryocyte count was 56/*μ*L. A urea breath test for HP was positive. The patient was diagnosed with ITP. HP was eradicated with 400 mg of clarithromycin, 1,500 mg of amoxicillin hydrate, and 20 mg of rabeprazole per day for 7 days. The patient's platelet count gradually increased and within 5 months had normalized. The patient's platelet count has remained within the reference range for over 2 years.

### 2.2. Case 2

A 75-year-old woman received an influenza vaccination; her platelet count measured the same day was 251,000/*μ*L. Five weeks after the vaccination, she was referred to our hospital because of thrombocytopenia with purpura combined with gingival and nasal hemorrhaging. Her laboratory data at admission were as follows: platelet count, 5,000/*μ*L; white blood cell count, 10,600/*μ*L without atypical cells; hemoglobin concentration, 15.9 g/dL; and PAIgG, 3780 ng/10^7^ cells. Bone marrow was normocellular. A urea breath test for HP was positive and a diagnosis of ITP was established. She experienced massive bleeding because of marked thrombocytopenia; hence, we started HP eradication as well as steroid treatment (1 mg/kg of prednisolone per day). Four weeks after the initiation of treatment, her platelet count had increased to within the normal range, so the steroid treatment was gradually tapered off over 2 months. No evidence of relapsed thrombocytopenia has been seen for over 15 months.

### 2.3. Case 3

An 87-year-old woman who showed no signs of platelet reduction such as purpura received an influenza vaccination. Two weeks after the vaccination, she was referred to us because of genital bleeding and purpura. Her laboratory data on admission were as follows: platelet count, 2,000/*μ*L; white blood cell count, 9,200/*μ*L without atypical cells; hemoglobin concentration, 9.3 g/dL; and PAIgG, 950 ng/10^7^ cells. The bone marrow megakaryocyte count was 111/*μ*L. We diagnosed the patient with ITP and began treatment with 1 mg/kg of prednisolone per day. The steroid treatment resulted in a rapid increase in the patient's platelet count, so it was tapered off over several months. The patient exhibited no evidence of relapsed thrombocytopenia after treatment.

## 3. Discussion

These cases illustrate two facts: influenza vaccination caused ITP in three elderly patients, and HP eradication and/or corticosteroid administration may effectively treat postinfluenza vaccination ITP in elderly patients.

ITP is associated with several types of vaccinations. In previous studies, the risk of developing ITP increased after administration of measles-mumps-rubella (MMR), some together with MMR,****hepatitis A, varicella, and diphtheria-tetanus-pertussis vaccines in children and adolescents [12]. Adjuvants, which are compounds incorporated into vaccines to enhance immunogenicity, have been implicated in autoimmune/inflammatory syndrome induced by adjuvants (ASIA) [13]. Although thrombocytopenia can also be seen in ASIA patients, the association between ITP and influenza vaccination is still unclear. A number of case-control studies and case reports concerning postinfluenza vaccination ITP have been published (Table 1) [1–11, 14]. The Berlin Case-Control Surveillance Study of drug-associated ITP concluded that influenza vaccinations increase the risk of ITP. Twelve cases of postinfluenza vaccination ITP, including our three, have been reported. Features common to most reported cases include PAIgG elevation, time from vaccination to development of ITP, and treatment response.

All of our patients and most patients in previous reports had PAIgG elevations. PAIgG can detect both surface IgG and *α*-granule IgG following platelet lysis [15]. Vaccine-associated ITP is probably caused by molecular mimicry, which involves the activation of autoreactive B or T cells by peptides in the vaccine that exhibit structural similarity to antigens found on platelets [12]. Elevated PAIgG may be an antibody produced by molecular mimicry.

Two of our patients developed ITP within 4 weeks after influenza vaccination and the third within 5 weeks. In previous patient reports, postinfluenza vaccination ITP developed 4–26 days after vaccination. This variability may depend on the patient's influenza antibody status. Some patients may have preexisting antibodies from prior influenza vaccines, some may be mounting an anamnestic response to a previously encountered antigen, and others may be undergoing primary alloimmunization because they had no previous exposure to the antigen [16]. An anamnestic response occurs in 3–10 days. Primary alloimmunization requires at least 2-3 weeks. Therefore, postinfluenza vaccination ITP in elderly patients may occur within several days or 2-3 weeks after vaccination.

In a previous report, elderly patients with ITP had a response rate of about 60% and were likely to relapse after treatment (31.3% of patients relapsed) [17]. In previously reported cases of postinfluenza vaccination ITP, 8 of 9 patients made a full recovery (Table 1). All three of our patients also achieved platelet recovery and were stable after treatment. Therefore, postinfluenza vaccination ITP in elderly patients may respond to treatment.

MMR vaccine is associated with the development of ITP in children [18]. The clinical features are similar to those of postinfluenza vaccination ITP. Signs usually occur within 6 weeks after vaccination [19], and more than 90% of patients recover from thrombocytopenia within 6 months after diagnosis [20]. In contrast to post-MMR vaccination ITP, 7 of the 12 reported postinfluenza vaccination ITP patients were elderly (Table 1), possibly because influenza vaccinations are recommended for elderly patients. ITP is likely to be found in elderly patients because they exhibit bleeding symptoms more frequently [21]. Two of our three patients with ITP were admitted to the hospital with bleeding symptoms.

HP eradication, corticosteroid administration, or both may effectively treat postinfluenza vaccination ITP in elderly patients. Prednisone, IVIG, and IV anti-D are recommended general treatments for ITP patients with severe thrombocytopenia or bleeding [22]. The rate of treatment-related adverse events, such as IVIG-related acute renal failure and steroid-induced diabetes or infection, is higher in elderly than in young patients with ITP [21]. Our patients' platelet counts increased smoothly without the use of IVIG and with no adverse events. One patient achieved full recovery and long-term remission with HP eradication only. We treated two patients with corticosteroids because of their bleeding symptoms. Post-MMR vaccination ITP is associated with a shorter duration of thrombocytopenia and less severe bleeding than is primary ITP [20]. For patients with postinfluenza vaccination ITP as well as those with primary ITP, corticosteroids and HP eradication may be effective treatments. HP eradication may be an effective and safe treatment for elderly patients with postinfluenza vaccination ITP, especially those without severe bleeding.

In conclusion, while influenza vaccinations are useful for preventing influenza infections and their complications, clinicians should be aware that an association with ITP is strongly suspected.

## Figures and Tables

**Table 1:** Clinical findings of previous reported cases of postinfluenza vaccination thrombocytopenia.

Study design	Reference	Number of ITP patients	Patient characteristics	History	Duration of thrombocytopenia from vaccination	Platelet count (/*μ*L)	Elevation of PAIgG level	Treatment	Outcome
Case-control study	Jadavji et al., 2003 [1]	2	NA	NA	10 days 14 days	NA NA	NA NA	NA NA	FR c-ITP
	Garbe et al., 2012 [14]	3	NA	NA	NA	NA	NA	NA	NA
	Grimaldi-Bensouda et al., 2012 [2]	43	NA	NA	<12 mo	NA	NA	NA	NA

Case report	Kelton, 1981 [3]	1	38-year-old, male	COPD	12 days	32000	Positive	Steroids	FR
	Casoli and Tumiati, 1989 [4]	1	32-year-old, male	None	15 days	NA	NA	Steroids	FR
	Granier et al., 2003 [5]	1	72-year-old, female	None	8 days	3000	Positive	Steroids	FR
	Ikegame et al., 2006 [6]	1	19-year-old, female	ALL (post-BMT)	14 days	10000	Positive	IVIG, steroids, and eradication	FR
	Tishler et al., 2006 [7]	1	68-year-old, male	HT	14 days	3000	NA	IVIG and steroids	FR
	Mamori et al., 2008 [8]	1	75-year-old, female	AILD	7 days	5000	Positive	Steroids	FR
	Tsuji et al., 2009 [9]	1	79-year-old, male	DM	4 days	4000	Negative	IVIG and steroids	c-ITP
	Mantadakis et al., 2010 [10]	1	3-year-old, male	None	26 days	11000	NA	IVIG	FR
	Shlamovitz and Johar, 2013 [11]	1	50-year-old, man	None	4 days	<5000	NA	IVIG and steroids	FR
			81-year-old, female	HT	28 days	27000	Positive	Eradication	FR
	Nagasaki 2016	3	75-year-old, female	AP and LD	35 days	5000	Positive	Steroids and eradication	FR
			87-year-old, female	None	14 days	2000	Positive	Steroids	FR

ITP: immune thrombocytopenic purpura; NA: not applicable; COPD: chronic obstructive pulmonary disease; ALL: acute lymphoblastic leukemia; BMT: bone marrow transplantation; HT: hypertension; AILD: autoimmune liver disease; DM: diabetes mellitus; HL: hyperlipidemia; AP: angina pectoris; IVIG: intravenous immunoglobulin; FR: full recovery; c-ITP: chronic-ITP.
